# Digital Domination and the Promise of Radical Republicanism

**DOI:** 10.1007/s13347-023-00618-7

**Published:** 2023-03-11

**Authors:** Bernd Hoeksema

**Affiliations:** 1 Faculty of Philosophy Theology and Religious Sciences, Radboud University Nijmegen, Nijmegen, Netherlands; 2 iHub: Radboud’s Interdisciplinary Research Hub On Digitalization and Society, Radboud University Nijmegen, Nijmegen, Netherlands

**Keywords:** Neo-republicanism, Radical republicanism, Digital platforms, Structural domination, Constitutive domination

## Abstract

In this paper, I approach the power of digital platforms by using the republican concept of domination. More specifically, I argue that the traditional, agent-relative interpretation of domination, in the case of digital domination, is best supplemented by a more radical version, on which republicans ought to give priority to structural elements. I show how radical republicanism draws attention to (1) the economic rationales and the socio-technical infrastructures that underlie and support digital platforms and to (2) the forms of influence that are directed at cognitive dimensions, such as habituation and routinisation, which are particularly relevant for the power of digital platforms. These insights also imply that republicans have reason to favour a more structural response to digital platforms over more direct and individualised forms of control that fit with a ‘standard’ republican approach, such as securing exit options and requiring user consent.

## Introduction

One of the today’s most discussed phenomena of the digital sphere is the rise to power of digital platforms like Google, Facebook and Uber. Their influence exceeds far beyond the digital and generates discussions on the platform economy on the influence they have on public policy and effective government, and a variety of others social issues, such as polarisation of political discourse and data governance in healthcare.

The power of digital platforms also poses a risk of domination, which, in republican terms, refers to an arbitrary or uncontrolled power to interfere with another’s choices. Republican freedom, or ‘non-domination’, requires the absence of such arbitrary power (Pettit, 1997, 2013). Radical republicans, however, challenge this view of domination, which I call the standard view, primarily for its agent-based nature. They propose to augment or replace the standard view with accounts of *structural* domination, where it is not individual agents but structures such as economic markets or social hierarchies that are primary factors of domination.

In digital contexts, global digital platforms have power over their user’s choices, and the means of controlling these powers is often similarly agent-relative: much depends on user consent and direct and individual control by citizens. But is this agent-relative understanding sufficient in digital contexts? Is the risk of domination just about several powerful agents such as Google or Facebook, or are there issues with underlying socio-technological and economic structures? And is securing exit options or user consent—usual ways of controlling power—sufficient for controlling arbitrary power in digital contexts? This paper aims to help formulate an answer to whether the standard republican account of domination is sufficiently specified and developed to capture what domination by digital platforms looks like.

In doing so, I draw extensively from radical republicanism. I argue, first, that a more radical version of republicanism illuminates how domination in digital contexts is marked not just by powerful agents, but also importantly intertwined with underlying economic and socio-technological structures, which are important instruments of domination. Second, I argue that digital technologies are perfect mechanisms for shaping the norms and values of the digital sphere through habituation and routinisation, notions that are not directly captured by the standard understanding of domination. They reflect what Michael Thompson calls ‘*constitutive* domination’ (Thompson, 2018, 50), which denotes an influence that is not directed at changing specific choices, but at ‘cognitive as well as evaluative dimensions’ of citizens (p. 50), in order to maintain a status quo that is in the interest of large tech firms. Recognising the importance of socio-technological and economic structures and of the cognitive dimensions of constitutive domination allows republicanism to develop a fuller and more specified understanding of (and response to) domination by digital platforms than a strictly agent-relative version does.^1^

In the first and second section, I set out the standard republican account of domination and its radical cousin. In the third section, I briefly discuss some of the ways in which republicanism is used to formulate a response to digital issues such as surveillance, automated decision-making and online deliberation. In the fourth section, I make my case for supplementing the standard, agent-relative, view with a radical view in digital contexts in order to capture the structural and cognitive elements prevalent in digital domination. I conclude by drawing some conclusions regarding the kinds of preliminary responses to digital domination that republicans, based on the radical perspective, are bound to favour: going beyond securing exit options and other forms of individualised control, aiming instead for a more radical restructuring of the technology and governance of the digital sphere.

## The Republican Project

### Freedom as Non-Domination

In this section, I will briefly sketch the ‘standard’ republican notion of freedom and some of its implications. I also discuss how some radical republican scholars attempt to move beyond an agent-centric approach of domination, instead pointing towards structural elements of domination. Both will play a role in the analysis of domination in the digital sphere.

The characterising component of the (neo-)republican project is its conception of freedom as the absence of domination. Domination broadly refers to a subjection to the arbitrary will of another. In Philip Pettit’s terms, domination exists where someone has the capacity to arbitrarily interfere with another, or where they have an uncontrolled power to do so (Pettit, 1997, 2013). What it means for a power to be arbitrary or uncontrolled is not settled between republican thinkers, but one leading interpretation emphasises the role of democratic decision-making: power is uncontrolled if those involved do not have a voice in the way it is exercised. It is not according to their ‘terms’ (Pettit, 2013, 50). This is why a slave is dominated, whereas (insofar conditions of control obtain) an employee is not: the slave can be interfered with as their master pleases and they have no voice in the matter. An employee, at least in an ideal situation, has various ways in which they can impose their terms on the powers of interference of the employer. They are protected by employment laws and/or unions, they can terminate the employment situation (provided alternatives or a safety net are in place), and they might have ways to exercise control through worker councils or corporate procedures, or ultimately via legal procedures. As they are able to set the terms of interference, these interferences are not the result of domination.^2^

The concept of domination serves the republican idea of freedom, which is posited as an alternative to what Isaiah Berlin calls negative liberty (Berlin, 1969). Republican freedom requires not just the absence interferences, as negative freedom does. Instead it requires, on the one hand, the absence of uncontrolled *powers* of interference, even where these do not give rise to actual interferences. For example, a slave subjected to a master who does not exercise his power (because, say, they are well dispositioned towards the slave) is unfree nonetheless. On the other hand, interferences that have their source in a controlled power do not result in domination, and, as such, do not compromise freedom. This is why a democratic government (with an independent judiciary), over which citizens have—hypothetically—equally shared control, does not compromise freedom, even if laws and policy necessarily interfere with their choices.

According to Pettit, domination can occur vertically, when a government has uncontrolled power over its citizens, and horizontally, when citizens dominate one another (Pettit, 2013, 136). Governments must impose a social order on its citizen, which is just insofar it minimises horizontal domination. This provides ground for legislation in many spheres of life, in particular those that are vulnerable to domination. But governments themselves must not dominate their citizens either: government power itself must be controlled. This is where questions of legitimacy come into play, bringing with them various institutional requirements, such as the rule of law and a system of checks and balances (Pettit, 1997, 2012).

### Agent-Centric Domination and Radical Republicanism

Many republican scholars have approached the conception of domination as agent-centric, or agent-relative, as is the case with the historic example of a master and a slave (Pettit, 1997, 57; 2012, 73; Lovett & Pettit, 2009, 14; Costa, 2009, 442). The concern is with agents who wield dominating power, remaining agnostic with regard to the *source* of domination. Agents can either be individuals or organised groups. Other republican scholars have recently argued in line with what James Muldoon (2022) calls a ‘social turn’ in republicanism. Broadly speaking, this radical version of republicanism states that domination materialises as part of broader structures (economic, social, institutional, etc.). In this section, I will sketch this social turn, and later, I will argue that a move to a more radical version is relevant for a better understanding of domination in the digital sphere.

According to the ‘standard’ republican account, domination eventually takes shape through (group) agents. An agent can wield dominating power by virtue of a broad variety of sources, such as (but not limited to) ‘certain legal advantages, more physical prowess, or greater social clout’ (Pettit, 2013, 62). A master dominating a slave, for example, is likely to have this dominating power as a result of a legal system of slavery and a corresponding social hierarchy, but a gunman dominating a passer-by does so by virtue of wielding a gun. Frank Lovett (2010) has us consider an island with a small number of slave masters who dominate a large group of slaves (p. 48). Lovett then argues that, were the slave masters to spontaneously repent and leave the island, the sudden absence of masters means that there would be no further domination. While the ‘ex-slaves’ may experience continuing harmful effects, for example malnourishment, the ex-slaves are no longer dominated because there are no agents with arbitrary power to interfere. Lovett’s example means to show that domination only exists within the interpersonal relations between agents, and that the underlying structures do not dominate as such.

Some republican scholars, often inspired by the nineteenth century labour reformers and later emancipatory movements, have criticised this agent-centric view or have proposed to augment it. Broadly speaking, they argue that by adopting an agent-centric view of domination, we risk overlooking the often *structural* aspects of domination, so they propose to move towards what has already been referred to as a radical version. Radical republican authors have generally focused on two areas of domination to establish their arguments: markets, capitalism and labour on the one hand (see, for example (Gourevitch, 2013; Thompson, 2013, 2018; Rahman, 2017; O’Shea, 2020; Muldoon, 2022) and social injustices, such as racism and sexism, on the other (e.g. Laborde, 2008; Coffee, 2015, 2020; Gädeke, 2020; Hasan, 2021).^3^ This second category shares much with feminist perspectives on power and domination. Iris Marion Young, for example, in the well-known ‘Justice and the Politics of Difference’, considers the concepts of domination and oppression to ‘primary terms’ in the approach to injustice (Young, 1990, 3). Her analysis finds its way into later (criticisms of) republican analyses of systemic social injustice (see, for example Krause, 2013, 201 and Hasan, 2021, 8 and footnote 22.)^4^

For the sake of brevity, I focus on the arguments made by radical republicans who are concerned with economic domination or domination in the workplace. Their analyses should suffice for the purpose of this paper. As mentioned, they more or less suggest that an agent-centric view of domination does not provide enough room for a full understanding of the forms of unfreedom prevalent in modern society: unfreedom that is the result of market mechanisms, economic unfairness and unequal control over productive assets within society (mirroring arguments against the patriarchy or systems of male or racial domination). Alex Gourevitch (2013), for example, shows how an agent-centric conception of domination only recognises domination insofar it is the result of *particular* masters. As such, while it can justify some version of universal basic income (in order to secure exit options exist for employees), it must leave intact the structure of the market, where some have better access to productive assets while others are always dependent on the labour market.

Instead, Gourevitch argues, we must understand that there are two different relations of domination: one interpersonal and one structural. Slaves in ancient Rome, according to Gourevitch, were not just dominated by a particular master, but also by the more ‘structural’ (p. 601) ‘many masters’, who maintained the legal and social institutions that kept slaves confined to their position more broadly. Similarly, Gourevitch suggests, if one group of owners has control over all productive assets in society, then non-owners have little choice but to sell their labour to *some* employer. They may ‘assent’ to voluntary labour, but they do not consent (p. 603, quoted from (Oestriecher, 1987). In such cases, whether or not a specific employer has arbitrary power to interfere with a labourer, the labourer is dominated structurally.

Michael Thompson (2018) suggests that we should think of domination along two dimensions: one extractive and one constitutive (the first being agent-relative and the other more diffuse). Both of them have to do with social structures. Extractive domination refers to a relation between two or more agents that exist for the purpose of extracting a surplus value from one agent to another. Thompson invokes the examples of capitalists exploiting workers by extracting labour, or men extracting benefits from women. These relations, Thompson notes, are hierarchical-structural. The domination is shaped by social norms, and institutions can even find its way into legislation, giving it a perceived legitimacy. But the hierarchical structures exist to benefit those at the top at the cost of those at the bottom.^5^

The other dimension of domination, Thompson suggests, is constitutive and refers to a control over values and norms that determine the ‘logics of culture and social institutions’ (p. 50). It is not so much a direct control over actions as it is a more subtle power over the consciousness of individuals, who through value pattern orientation, habituation, routinisation and cultivation can be made to accept and internalise certain social relations, institutions or authorities as legitimate. What separates, according to Thompson, constitutive domination from normal, non-dominating social reproductions is that the first contains a hierarchically organised select group that shapes the norms and institutions of a society *in their own interest*. It is a power to ‘(i) shape social norms, (ii) routinise values and value orientations, (iii) orient consciousness and the cognitive and evaluative powers and patterns of subjects, and (iv) legitimate extractive relations’ (p. 52).

Other radical republicans are similarly concerned with the danger of overlooking impersonal domination. Keith Breen suggests that we should not focus merely on contractual inter-agent relations, but on the ‘hierarchical governance structures of productive enterprises’ (Breen, 2017, 425). He concludes that exit options are not enough to guarantee non-domination in the workplace, but that workers should have a sufficiently controlling voice within enterprises. Sabeel Rahman argues that (economic) domination can be both inter-agential and structural, where the first brings to mind managerial or corporate power and the latter refers to domination that is the result of the ‘market system itself’ (Rahman, 2017, 42).

What these and other radical republican scholars share is that they draw attention away from agent-centric forms of domination towards a concern for structural and diffuse domination, which they argue justifies more ambitious institutional reforms than republicans usually support. Instead of securing exit options for employees, for example, one needs to transform the culture of work (Gourevitch, 2013), ensure strict state regulation and promote democratisation of the workplace (Breen, 2017). In more general terms, in order to have a convincing ideal of social freedom, the ‘structure as well as purposes and goals of any society need to be transformed’ (Thompson, 2018, 52).

### The Strengths of a Republican Approach

Let me conclude this sketch of republicanism, both the standard and its radical turn, by explaining why republicanism and its radical version offer a good starting point for an investigation of freedom in the digital sphere. First, republicanism offers a complete and comprehensive political doctrine which offers both tools for analysis of dynamics of (un)freedom and for formulating institutional responses to them. A second reason comes from the conceptual link republicanism draws between freedom and power, which fits neatly onto the often voiced unease regarding the powers of and dependency on ‘Big Tech’. It acknowledges that the mere capacity that technology provides to agents can be enough to alter the balance of control and power, which are tied to domination and loss of freedom. It also incorporates the uncertainty that is the result of knowledge asymmetries between users and powerful organisations employing technology.

While the republican concern for freedom goes beyond actual interference, the idea of non-domination also allows for desirable *controlled* interference, without conceptual loss of freedom. With a negative understanding of freedom, interferences by tech companies and governments must be considered sacrifices to our liberty, as if a degree of freedom should be traded away in order to help us navigate the internet, select preferences and filter irrelevant or harmful information. By contrast, on a republican account, such interferences could be compatible with (or even contribute to) freedom, insofar they are bound by terms set by those subjected to them. The republican project implores us to focus on the more constructive question of control: to what extent *do* people have control over powers of interference that they face in their online activities, and to what extent do they have control over the structures (and infrastructures) that allocate these powers?

## Digital Domination

With the conception of freedom as non-domination in hand, we can show how technology, in general, might create or increase domination of citizens. Technology provides individual and group agents, such as tech companies and governments, with new (or increased) capacities to interfere on an uncontrolled basis. To flesh this out, I discuss some important examples of areas of domination in the digital sphere according to the standard version of republicanism.

### Data Collection and Surveillance

Ever since the Snowden-revelations, scholars and activists have voiced concerns regarding technology-enabled mass surveillance and breaches of informational privacy and autonomy in the digital sphere, and some have done so from a republican perspective. Newell (2014) and Roberts (2015) explore how breaches of privacy and use of surveillance can constitute domination, not only because it enables uncontrolled interferences, for instance by allowing others to remove or change options based on sensitive personal information, but also by taking away the choice of others to share or hide information in the first place. In addition, they show how the notion of domination is well equipped to tell us why surveillance even *without* actual interference may compromise our freedom and autonomy: our control over who has access to our information and what it is used for with it is limited. On a republican account, it does not matter whether we are aware of interferences occurring or not. In fact, it is not knowing whether we are watched that might give us an uneasy and uncertain feeling, and the idea of domination captures this dynamic well. We cannot know for sure whether interferences have occurred, but we know that others have *the capacity* to do that. It is only when this capacity of surveillance is subject to citizen’s direct or indirect control that we are free from domination. In fact, minimising domination might in fact require forms of controlled surveillance (Smith, 2020).

### Choices and Algorithmic- and Automated Decision-Making

Domination might also materialise in the use of algorithms or other forms of automated decision-making (Danaher, 2020; Gräf, 2017). Eike Gräf (2017) argues that automated profiling can be used to interfere with individuals in various ways. It can be used (1) to decide about them, (2) to decide not to engage with them, (3) to shape their choices, (4) to limit their options and (5) to replace their options. These interferences allow agents who have an uncontrolled capacity to use automated profiling to dominate individuals in pervasive ways, although the severity depends on the amount of power. An uncontrolled power of an app or website to suggest new songs for consumers, for example, seems to have little impact. Yet we clearly have reason to fear an uncontrolled power of a real estate website to exclude whole neighbourhoods based on the racial profile of its visitors, for its severe individual and societal impact and for its discriminatory nature.

John Danaher sees a similar risk in the use of algorithms in governing people, for which he uses the term *algocracy* (Danaher, 2020). The term refers to ‘the unavoidable and seemingly ubiquitous use of computer-coded algorithms to understand and control the world in which we live.’ (p. 2). In such a society, algorithms nudge and coerce our daily or benign choices. And while we may think of many of these powers of interference as insignificant, together, they could amount to ‘micro-domination’, referring to the idea that an aggregate of seemingly insignificant powers of interference may nevertheless render one subject to significant domination (Danaher, 2020; O’Shea, 2018). Similarly, in an algocracy, individuals might be subject to ‘algorithmic masters’ by virtue of the many small, daily choices in which they are dominated (Danaher, 2020, 23). Consider, for example, how someone doing groceries might be dependent on pervasive and tailored algorithms for navigation, dinner and brand choices, product selection, etc. If this use of algorithms to understand and interfere with the choices of others is uncontrolled, we risk living and choosing by the leave of the algorithms to which we have subjected ourselves.

### Democracy and Social Media

A final example of a republican critique of the digital sphere is that of speech on digital platforms. Ugur Aytac (2022) highlights how social media companies have the power to regulate communication on their platforms, amounting to what he sees as a particular form of domination. Aytac considers two variants. First, citizens are subject to *direct* arbitrary interferences with their online speech. The firms that control these platforms, by virtue of the dependency of users, have the power to interfere with public speech by prioritising information and communication. A second form of domination is the result of social media companies having *indirect* control over the algorithms that govern the digital sphere. This, according to Aytac, gives them the power to determine the modes of engagement that citizens have access to (which can be either deliberative, antagonistic or mixed). As social media companies attempt to maximise engagement, they may ‘systematically incentivise uncritical, one-sided, and reactive online behaviour’ (p. 12), implying a shift in the available modes of engagement. This is what constitutes the second version of this domination over communication: social media companies have an arbitrary power to interfere with the modes of engagement available to citizens.

## Beyond Tech Giants: Radical Republicanism in the Digital Sphere

Most of these accounts of domination in digital contexts use the standard republican approach of domination, that is, they are primarily concerned with agent-centric domination.^6^ As Aytac (2022) argues: ‘the powers of social media companies are rather centralized and under the intentional control of corporate bodies’ (p. 3). Indeed, many—if not most—threats of domination in the digital sphere seem to radiate from powerful corporate agents such as Google, Meta or Twitter. In this section, I argue that it is valuable to set loose the radical republican concerns on digital platforms in order to capture the distinctive, diffuse and structural sources and dynamics that make up the digital sphere, such as its connection to underlying economic systems and the prevalence of subtle online manipulation.

Let me first clarify—again—that in doing so, I do not mean to argue against the ‘standard’ republican account, nor attempt to establish that the radical view does things that a standard account cannot. I also do not suggest that domination is *necessarily* structurally constituted. Instead, I take an ecumenical approach, allowing domination to exist in both agent-centric *and* diffuse and structural forms. Republicanism can acknowledge the existence of particular (group) agents who dominate others while recognising the need to go beyond—towards the structural dynamics that mark domination in the digital sphere. With this ecumenical approach, one can acknowledge the problem of uncontrolled powers of specific online platforms like Google and Twitter, while also being concerned with the socio-technological and economic structures from within they operate.

From the radical republican perspective, I suggest one can draw at least two important insights with regard to the dynamics of domination by digital platforms. The first is that republicans should evaluate and, where necessary, change underlying economic and socio-technological background structures and infrastructures to reduce (structural) domination. In fact, they should give *priority* to structural domination over agent-relative forms. The second insight is that there is an important cognitive element to the dynamics of domination on digital platforms: power is often exercised through more subtle, manipulative practices and through habituation and socialisation. Although this concern is not new, these practices are particularly associated with digital platforms, which are in a position to use this influence to shape the digital sphere in their interest. This concern deviates somewhat from the coercion-based paradigm that is often used in republicanism. These insights are not just conceptual, but will prove important factors for determining the strategy that one should adopt on a republican account.

### Highlighting the Importance of (Infra)structural Domination in the Digital Sphere

A few large online platforms, owned and maintained by a few large technology companies (often referred to as Big Tech), have become dominant players in much of the digital realm and large parts of our offline lives that depend on that. Yet, it would be a mistake to approach their dominance as an issue that can be understood or countered without also approaching the (infra)structures that are both the result and the enabling factors of their dominance. Their position of power that did not simply ‘come to’ exist, but is one that is arguably the result of deliberately exploiting a *tendency* of concentration of power that marks the digital sphere.

Digital platforms, while their services and business principles differ, share certain important characteristics that set them apart from other firms, and which help explain the particularity of their power. Nick Srnicek (2017, chap. 2) identifies several of these. First, digital platforms can be thought of as intermediary *infrastructures* that allow various other parties to interact. Second, digital platforms make extensive use of a network-effect: the more users are active on a digital platform, the more valuable it is. Third, platforms might make some parts of their services or products free, while raising prices in other sections. In this way, they promote their services and increase data collection at a cost, while making a profit in other ways: profits and costs are not equally distributed over a platform. Finally, digital platforms determine, through the design and governance of their infrastructure, the rules and possibilities of the interactions that take place.

To get to into that position, digital platforms first need to grow towards occupying a dominant position. It is the scale itself that is a prerequisite for success, which signals a corresponding change in the dynamics of investment. Investors are more patient, allowing a firm to pursue its promise of market domination (Rahman & Thelen, 2019). It is only in the second stage of the digital platform model, when the platform has taken its role in the new socio-technical landscape, that returns are expected (Hendrikse et al., 2022). This is because scale is critical to their ability ‘to cultivate and capture value’, as it allows them to monetise the platforms and computational infrastructures they then come to control and that others come to depend on (Langley & Leyshon, 2017, 22). The economic rationale can be considered to be a new form of ‘rentiership’, where data and infrastructures are privatised and used to make economic rents, which ‘reinforce [Big Tech’s] techno-economic power, while undermining the political, social and economic capacity of others to shape the future’ (Birch & Cochrane, 2022, 53).

The result is that digital platforms operate on a the-winner-takes-it-all logic, where winning reflects not just a large market share, but significant control over digital and material infrastructures and over further innovation. This is clearly visible in, for example, the advances in artificial intelligence, where large technology companies control most of the datasets, expertise and computational resources required to develop and leverage AI (Verdegem, 2022) or in behavioural modification research, where researchers have trouble accessing data in possession of digital platforms required for academic purposes (Greene et al., 2022).

Digital platform power, thus, is not a power that primarily reflects the ability to coerce by force, by some legal authority, or that is limited to through direct interaction with users on platforms, but one that reminds of the power wielded by public utility companies. Citizens and governments (have) come to depend on the infrastructure they control (Rahman, 2018).^7^ This is particularly immanent where the dependency is more fundamental to the user or supplier, as is the case with (delivery) drivers, content creators and others who depend on the platform for their daily wages. On the user’s end, the problem may seem smaller, but there too users often (and increasingly) depend on platforms for important social interactions (in the case of social media platforms), career opportunities (in the case of LinkedIn) or, as discussed above, for political deliberation.

Importantly, this dependence also extends to public systems. As part of the response against COVID-19, many governments in the European Union relied on infrastructure provided by Google and Apple for the rollout of Covid-19 contact tracing apps (Lanzing et al., 2022; Sharon, 2020). But also in sectors other than healthcare—security, education, law enforcement and so forth—governments are increasingly relying on digital infrastructures, which raises issues of intermingling of public and private values (L. Taylor, 2021). The ‘Sphere Transgression Watch’, for example, is a tool that aims to visualise how large technology companies have, over the course of a decade, started to spill over into other public spheres, including education, the environment, agriculture, security and mobility (Stevens et al., 2022).

The dependence on their control over infrastructure already provides digital platforms with significant power, which is further strengthened by the direct and unmediated connection platforms have with their users. Governments, wishing not to provoke consumers, have previously been reluctant to interfere extensively with firms that provide the services and amenities that so many citizens depend on and use every day (Culpepper & Thelen, 2020; Rahman & Thelen, 2019). This power is enabled by countless of users who have come to depend on the infrastructure and services these companies provide. The so-called cookie-directive (Directive 2002/58/EC, 2002) might serve as an example of this tension, where many users perceive attempts to protect their privacy in the form of cookie consent pop-ups as annoying interruptions of their online activity, a fact that firms happily emphasize and exploit in order to make regulation unpopular. Culpepper and Thelen (2020, p. 306) discuss several cases where this power has allowed large tech companies to successfully mobilise their userbase in political campaigns to their own advantage.

There is one further point to make here: the values and rationales of the companies behind the platforms shape the technology itself. Privacy-defaults, dark patterns and nudges are used to steer users to act in the interest of the designers (Grassl et al., 2020), and the—often default—centralised storage of data is accompanied by centralised control (Jacobs, 2010), further strengthening the structural positions of power. The possibilities and parameters of the software and the design of the underlying architectures determine and reinforce, for a large part, the dynamics of power (Muldoon & Raekstad, 2022). The material ideology is built in the product designs and policies of tech firms (West, 2019). Conversely, technologies cannot be understood without their broader socio-technological context (Crawford, 2021; Kitchin, 2017).

All of this drives home the point that the power dynamics of digital platforms must be understood as deeply embedded in social, technological and economic structures. A standard republican account of domination is able to tell us how digital platforms enable agent-relative forms of domination. But similar to how radical republicans approach domination on the labour market as a systemic issue, so a radical version draws out how the economic rationales and socio-technical infrastructures work together to create a self-reinforcing system that makes citizens and governments alike heavily dependent on the control of digital platforms. James Muldoon and Paul Raekstad (Muldoon & Raekstad, 2022), for example, concerned with the gig-economy, have already drawn from radical republicanism in developing an account of algorithmic domination, and their understanding ‘emphasizes the social relationship and structural power inequalities at the heart of the system’ (p. 7). They suggest that their account of algorithmic domination can be applied to the labour market in general and to other domains.

This emphasis on structural elements that radical republicanism brings is relevant for a broader digital context. It makes little sense to curb the power of large tech corporations by securing exit options and consent, without giving priority to underlying (infra)structural issues such as the large asymmetries of knowledge and resources, and the tendency of platforms to monopolise and to control infrastructure. As we shall see, that comes with implications for the kind of institutional responses that republicans should favour.

### Highlighting the Importance of Constitutive Domination in the Digital Sphere

This brings us closer to a second insight drawn out by the radical perspective. Although the republican concern with interfering power is not strictly limited to open and coercive forms of interference—Pettit acknowledges that manipulation can also fit the bill (Haugaard & Pettit, 2017; Pettit, 1997)—domination is generally thought of as being ‘common knowledge’ (Pettit, 1997, 60). Indeed, most paradigm examples of domination—slavery, certain forms of traditional marriage and employment—seem to be largely coercive in this sense. The dominator can, for example, explicitly draw attention to their position of uncontrolled power: ‘I am your master [husband, boss, …], so you do as you’re told (or else…)’.

However—in addition to the dependency on platforms outlined in the previous section—the influence of digital platforms is often akin to subtle manipulation rather than to outright coercion or threat thereof. Influence is quite often aimed directly at the cognition of citizens. This is exacerbated by a general transparency and knowledge asymmetry that exists between individual users and digital platforms, which invested heavily into gathering and analysing data on user behaviour (Aho & Duffield, 2020; Greene et al., 2022). The mechanisms and policies behind digital platforms are generally opaque and their activities covert (Crain, 2018; Zuboff, 2019; L. Taylor, 2021).^8^ That does not apply to their users, whose data is up for grabs by many digital agents that use their data to make extensive profiles and research user behaviour. Privacy notices, formally meant to inform, are often long and complex and require too much time and cognitive load for most users to read (McDonald & Cranor, 2008; Veltri & Ivchenko, 2017; Obar & Oeldorf-Hirsch, 2020). The underlying ideal of rationally consenting consumers is undermined by technology firms making services dependent on consent or by the use of dark patterns to nudge them to quickly accept (Grassl et al., 2020; Nouwens et al., 2020). In fact, users may have gotten used to quickly consent in order to proceed with their intended activities (Böhme & Köpsell, 2010).

These asymmetries of knowledge and resources allow digital platforms to exercise influence over users through powerful and hidden manipulative practices, such as designing choice-environments, rather than through explicit coercion (Viljoen et al., 2021). This is the source of a broader concern that has gained further traction since the Cambridge Analytica scandal in 2016: that of widespread and routine online manipulation. Although previously many scholars focussed on particular harms of online manipulation, there is increasing concern for the broader impact on the digital sphere. Daniel Susser, Beate Roessler and Helen Nissenbaum identify some reasons why digital technologies are particularly effective in mediating widespread manipulation. First, information technology ‘puts our decision-making vulnerabilities on permanent display’ (Susser et al., 2019, 6). Online, it is substantially easier to gather information on the behaviour of users, both on an individual level as well as on a larger scale. Second, digital platforms are perfect media for using that information to press vulnerabilities and meddle with decision-making: they are automated, real-time, consistently present and very personalised. Third, the way in which we think of and use technology ensure that such efforts are always, in a sense, ‘hidden’. We are, usually, not concerned with the way in which technology mediates the way in which we use it. Technology is, in a way, ‘invisible to us through frequent use and habituation’ (p. 7).

Although Susser, Roessler and Nissenbaum do not go as far, they note that others have implied that these practices are so characteristic of the digital sphere that they, in a way, constitute it. This reflects a rapidly growing body of literature that critically evaluates the digital sphere through concepts like *data capitalism* (Sadowski, 2019; West, 2019), *platform capitalism* (Srnicek, 2017; Viljoen et al., 2021), *AI capitalism* (Verdegem, 2022) and perhaps most famously, *surveillance capitalism* (Zuboff, 2015, 2019). Many of these take traditional critiques of capitalism and apply them to the digital sphere, in which they recognise capitalism’s ‘next steps’. What is especially interesting to note is that such accounts are often sensitive to more subtle mechanisms of power, such as powers of socialisation and habituation. Shoshana Zuboff, for example, is repeatedly concerned with the suggestion that as more and more of our lives are subject to surveillance capitalists, ‘we lose our bearings as institutionalization first establishes a sense of normalcy and social acceptance, and then gradually produces the numbness that accompanies habituation’ (Zuboff, 2019, p. 277). By aggressively shaping the digital sphere, and by ignoring first waves of critique and resistance, in other words, they habituate individual and public perceptions of the status quo.

The subtle mechanics of power are not unknown to radical republicans. They bring us back to Thompson’s version of domination, which explicitly incorporates these dynamics in constitutive domination. Thompson equips republicanism with the tools to recognise a power over consciousness, through the habituation and cultivation of norms and values. A subtle power that is not directed at changing specific choices, as outright manipulation and coercion do, but a power that might be used to make citizens accept and internalise things as ‘given’, including the way in which the digital sphere is structured and presented to them.

Applied to digital platforms, the concern is that these platforms are not just potential agent-relative dominators, but also dominating in this constitutive sense: their central positions allow them to shape the norms, values and institutions of the digital sphere *in their own interest*. This also helps explain how large digital platforms beat new competition: not only do they merge with them, they also ‘shape imaginaries of what innovation could look like — not, precisely, by direct domination in the market, but through its cultural influence on the gestation of entrepreneurs’ ideas’ (Hellman, 2022, 156). Accommodating this form of power in the republican framework has distinct advantages over a focus limited to agent-relative, choice-based forms, in particular in the digital sphere. It could, for example, help understand how citizens may develop a certain deference or lethargy with regard to forms of digital domination. Citizens could come to think of them as disruptive business actors, legitimately exploiting users for private gain or as a necessary component of effective public governance. Digital platforms, as hidden media, are perfect tools for such forms of domination.^9^

## Conclusion and Some Implications for Responding to Digital Domination

I conclude that agent-relative forms of republicanism, with a focus on arbitrary interference by powerful agents, can be supplemented with radical versions of republicanism, which emphasise the role of structural and constitutive domination. The first refers to how underlying (infra)structures result in digital platforms occupying positions of power, while citizens and governments are increasingly rendered dependent on their control. The second refers to a power that is not directed at choice but at changing the norms and values that shape the digital sphere through habituation or routinisation. These seem especially relevant in digital contexts, which cannot be seen apart from their particular economic and socio-technological rationales and designs, and which are perfectly suited for widespread, subtle forms of manipulation, socialisation and habituation. A comprehensive republican approach to digital domination, which has so far mostly focused on the role of powerful technology companies, benefits from incorporating this emphasis on (infra)structural and cognitive elements, or sources, of domination.

Apart from illuminating dynamics particular to digital domination that on a standard account of domination may be overlooked or taken for granted, the insights drawn out by taking a radical perspective also come with implications for institutional responses to digital domination. Republicans have traditionally proposed various ways in which one can realise control over arbitrary power, and the radical perspective implies that there are some that are more preferable than others. The task is to adapt this radical republican account to the issues raised above. I cannot give a full account here, but there are several implications that give an idea of what this would look like.

The first implication is that republicans cannot rely on the idea of exit options or of rationally consenting citizens. Traditionally, republicans have recognised exit strategies as a viable way of mitigating arbitrary power—or rather as a way of escaping it. This has led to an ambivalent view on the role of the market, which would, if exit options are secured, allow for a non-dominated space (R. S. Taylor, 2019). In this vein, many regulatory approaches to the power of big tech aim to provide users with a set of rights, including rights to give and revoke consent and to be provided with certain information (Susser, 2019). Radical republicanism casts doubt on that idea in two ways. First, taking seriously the risks of constitutive domination, users might quite easily be socialised, habituated or manipulated to simply accept the status quo, including the need to consent in order to access certain services. The second reason is that, as we have seen, the power of digital platforms flows largely from their control over resources, expertise and computational infrastructure, which increasingly extends not just towards important aspects of daily life, but also towards public systems. So even if citizens were consistently able to see through a choice-environment skewed against them in ‘market-like’ environments, then they likely would still be subjected to inescapable government systems—bringing them back into the sphere of influence of those same platforms. In short, on a radical republican account of the power of big tech, there is little place for reliance on ‘notice-and-consent’ mechanisms or other exit-strategy–based responses. In the case of (infra)structural dependency, we may be reminded of Gourevitch’s point on the impossibility of consent: users, in some cases, they may, at best, *assent*, rather than consent (Gourevitch, 2013, 603).

A second implication that flows from adopting a radical republican perspective is that responses to domination by digital platforms must include some effort to empower citizens to withstand socialisation and habituation. This warrants taking a closer look at the behavioural research done by digital platforms. Rather than expecting users to fend for themselves—or to exercise their rights—it implies the need for strict regulation of online manipulation in various forms, for dedicating sufficient resources to educating citizens, and for providing access to the data and research done by digital platforms on behavioural modification (Greene et al., 2022).

For a third implication, we turn back to the increasing influence of large tech platforms within public systems, through the various mechanisms discussed in this work. This has a diffusing effect on the difference between private and public and the values that govern both, and also on the republican idea that one could separate state domination (*imperium*) from private domination (*dominion*) (Pettit, 1997). Government bodies and public officials must be aware of this, and they share a duty to curtail the undermining effect of a reliance on private digital platforms for the public tasks they are entrusted with. They should, for example, ensure that they gather information from various credible sources, rather than basing decisions merely on information provided by large technology firms (Meghani, 2021). Government must explicitly be held responsible with regard to the broader ways in which the intermingling between private digital platforms and public organisations affects citizens (L. Taylor, 2021).

A final, more general implication is that any republican response to the power of digital platforms must reach far enough to evaluate and alter economic rationales, socio-technical structures and computational infrastructure in order to root out as much of the dependency on powerful, private digital platforms as possible. One way of doing so is to promote a ‘digital commons’, an idea that has gained traction over the years (see, for example: Collins et al., 2020; Fuchs, 2020; Verdegem, 2022). This notion promises, roughly speaking, a communally owned and governed digital sphere to which all citizens have access. A digital commons gives citizens a voice in the structures of the digital sphere and allows them to escape both the domination by particular platforms as well as the structural domination ‘by many platforms’. It could also contribute to a fair distribution of the resources and expertise needed for profiting from new technologies. This approach warrants regulating companies that offer (increasingly) vital services in ways similar to public utilities companies, and it calls for active government support for developing open-source and publicly owned alternatives. Both might need to extend to the material level, where even computational infrastructures are brought into the commons in order to prevent dependency on existing, privately owned systems.

These suggestions have been recurrent in existing literature on digital issues. This paper, however, shows how republican political theory, in particular when supplemented by a radical perspective, is able to offer a comprehensive, strong justification for suggestions in line with the ones just discussed. On a radical account, preventing online domination requires more than securing exit options, or restricting powerful digital agents in their capacity to (arbitrarily) interfere—it might call for a radical restructuring of the digital sphere and the platform mechanisms that shape it.

## Data Availability

Not applicable.

## Footnotes

1 The aim of this paper is not to argue that the standard version of republicanism is conceptually *unable* to incorporate insights from radical republicanism. Philip Pettit, for example, recognises the significance of naturalised forms of power, which can encompass routinisation and habituation and which may lead to agent-relative domination (Haugaard and Pettit, 2017). Similar arguments might be made for other radical republican concerns. For the purposes of this paper, however, it is not necessary to commit to a standard version or a radical one. I draw from radical republicanism to discuss some dynamics of domination by digital platforms and to show that a focus solely on the power of specific agents or on securing exit options fails to capture some issues of digital domination. As such, I consider my efforts to be part of a broader republican project and not part of a specific interpretation.

2 Of course, many employment situations may fail to (fully) live up to the ideal of non-domination.

3 Note that radical republicans who are concerned with domination in economic structures are still concerned with liberty (and the impact of markets thereon), not with the quality of markets *as such* (e.g. lack of competition).

4 The master–slave paradigm has also been used to draw out systems of subordination of women to men. See, for example, Marilyn Frye (1984, 103–4).

5 And, as such, differs from *authority*, where extraction is not the point of the relation. This is why relationships between parents and children and between teachers and students, which are also embedded in social structures, do not amount to extractive domination.

6 A notable example is a recent paper by James Muldoon and Paul Raekstad (Muldoon & Raekstad, 2022), in which they discuss algorithmic domination in the gig-economy. They develop the concept of algorithmic domination and apply it to the gig-economy, and they recognise the mediative power of digital platforms and their socio-technological background. They draw explicitly from labour republican scholars like Gourevitch and Thompson. In this paper, my concern is more abstract: I aim to show the strengths of radical republicanism to analyse digital platforms more broadly.

7 As such, we have reason to regulate such monopolists as if they were public utility companies.

8 Implying the need for a duty of care for those parties that are in a position of more knowledge and expertise.

9 Philip Pettit seems to be open to the idea that social norms and institutions, insofar they lead to dyadic domination, can be ‘naturalised’ in the minds of individuals, and, as such, can be difficult to combat (Haugaard and Pettit, 2017). A standard account of domination does not exclude the concern for domination through a power over consciousness beforehand. A radical account nevertheless seems to be more focused and critical on such forms of power and does not need to trace it back to specific agent-relative situations.
